# Thyrotoxic Periodic Paralysis Causing Back Pain and Leg Weakness: An Unusual Presentation of Hyperthyroidism

**DOI:** 10.1155/2021/6622658

**Published:** 2021-03-16

**Authors:** Henrik Elenius, Marie Cesa, Corina C. Nava Suarez, Abhishek Nimkar, Prasanta Basak, Nandita Sinha

**Affiliations:** Montefiore New Rochelle Hospital, 16 Guion Pl, New Rochelle, NY 10801, USA

## Abstract

Thyrotoxic periodic paralysis (TPP) is a rare muscular disorder, characterized by muscle weakness and hypokalemia triggered by thyrotoxicosis. In Asian populations, 2% of patients with thyrotoxicosis are affected, compared to only 0.1–0.2% of non-Asians. The vast majority of patients are male. Muscle weakness ranges in severity from very mild to life-threatening, due to respiratory compromise. We present a case of a previously healthy 39-year-old Hispanic male who presented with sudden quadriparesis and quickly recovered after being treated for hypokalemia and thyrotoxicosis. TPP, although unusual, is important to recognize as it is a potentially fatal condition that requires close monitoring and is readily reversible with appropriate therapy. Any cause of thyroid hormone excess can cause TPP, with Graves' disease being the most common etiology. Acute treatment includes potassium repletion, while long-term management focuses on determining and treating the cause of thyrotoxicosis, since maintaining a euthyroid state will prevent further episodes of TPP.

## 1. Introduction

Periodic paralysis is a rare group of muscular disorders characterized by episodes of muscle weakness in combination with hypo- or hyperkalemia. Most cases are familial, but a small subset of cases, designated thyrotoxic periodic paralysis (TPP), are acquired with thyrotoxicosis triggering episodes of paralysis and hypokalemia. While hyperthyroidism is more common in females, >95% of patients with TPP are male. TPP is also significantly more common in Asian populations, affecting 2% of Asians with thyrotoxicosis, compared to 0.1–0.2% of non-Asians [1]. Although unusual, it is important to recognize TPP, as it is a potentially fatal condition which is readily reversible with appropriate therapy.

We present a case of a 39-year-old Hispanic male with no past medical history who presented with sudden quadriparesis and quickly recovered after being treated for hypokalemia and thyrotoxicosis.

## 2. Case Presentation

A 39-year-old Venezuelan man with no past medical history presented to the emergency room with recurring, cramping pain in his lower back and legs, along with mild bilateral leg weakness for 1 month. These symptoms would return every afternoon, when the patient came home from his job in demolition, and then resolve by the following morning. Over the same period of time, he had also experienced intermittent palpitations at rest, hand tremors and excessive sweating. The day before presentation, his pain and weakness got significantly worse, causing a fall while walking. On the morning of presentation, he was unable to ambulate without support due to severe leg weakness. His lower back pain was described as sharp, 8/10 intensity, exacerbated by movement and radiating down his legs to both feet. Prior to his symptoms starting in the preceding month, he had never experienced similar episodes in the past. The patient also noted a 5 kg unintentional weight loss over 4 months. He denied any recent injuries, constipation, diarrhea, problems with urination, vomiting, recent travel, recent illness, new medications or supplements. He had no heredity for muscle or thyroid problems. He had never had any thyroid issues, and blood work as part of a routine check up had reportedly been normal 5 months prior, although these results were not available for review.

Vital signs were only notable for a heart rate of 98 beats per minute. Physical exam was notable for proximal weakness in all extremities, with the patient only being able to move his limbs against minimal resistance. Additionally, decreased range of motion was seen on bilateral hip flexion and right shoulder abduction owing to sharp pain on movement. Deep tendon reflexes were reduced in all extremities. Sensation was intact. Symmetrical hand tremors were noted with arms outstretched. Bilateral exophthalmos was noted. Cranial nerves were intact, and no diplopia was seen. Thyroid exam was unremarkable, without any enlargement, nodules or tenderness noted. No further abnormalities were noted on systemic examination.

**Figure 1:**
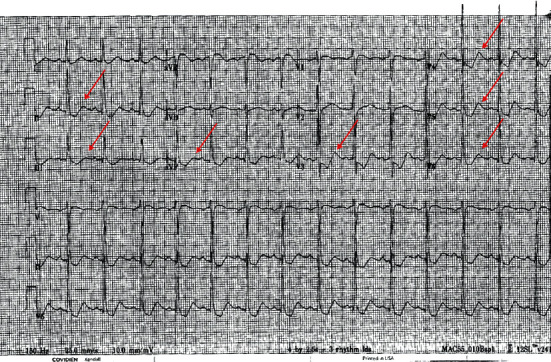
Admission ECG showing inverted T-waves followed by U-waves (red arrows) in leads II, III, AvF, and V3–V6, a sign of hypokalemia.

A CT of the lumbar spine was unremarkable, with no significant central canal stenosis or neural foraminal narrowing. Laboratory findings, reviewed in Table 1, revealed low levels of potassium, 1.7 mmol/L; magnesium, 1.3 mEq/L; and phosphorus, 1.5 mg/dL. A complete blood count and the remainder of the comprehensive metabolic panel were both normal. Thyroid function tests showed a low thyroid-stimulating hormone (TSH), 0.010 mIU/mL as well as elevated free T4, 2.98 ng/mL and total T3, 399 ng/dL. Further thyroid testing revealed elevated thyroid-stimulating immunoglobulin (TSI); 278% of baseline; thyroglobulin antibodies, 20.0 IU/mL; and thyroid peroxidase antibodies, 180.0 IU/mL. An ECG was notable for inverted T-waves followed by U-waves in leads II, III, AvF, and V3–V6 (Figure 1).

The patient was given a total of 90 mEq of oral potassium chloride on the day of admission. Magnesium and phosphorus were repleted as well. In addition, he was started on oral methimazole (40 mg, twice daily) and propranolol (40 mg, three times daily) on admission. By the next morning, his potassium level had normalized, and his weakness and back pain had resolved completely. He was discharged on beta-blockers and methimazole. After discharge, the patient struggled with medication compliance due to financial constraints. As of the last follow-up, three months after discharge, he had resumed his medications with reported resolution of all symptoms. He was planned to receive radioactive iodine therapy, but was eventually lost to follow-up.

## 3. Discussion

This case highlights TPP, a rare condition, especially in a patient without Asian heritage. TPP is characterized by episodes of sudden generalized weakness, with the age of onset being 20–39 in 80% of patients, unlike familial cases of periodic paralysis, which usually debut in childhood or adolescence. Symptoms of hyperthyroidism develop around the onset of TPP in around 25% of patients, but can also appear years earlier or later [2]. Our patient had symptoms for a few months prior to developing TPP, with weight loss, palpitations, heat intolerance and tremors noted. The muscle weakness in TPP is predominant in proximal muscles, with legs being more affected than arms. Decreased muscle tone with decreased reflexes is typical [3]. Myalgia is seen in around 40% of patients and can occur before or concurrently with the paralytic attack [4]. Episodes typically last from several hours to days. Apart from hypokalemia, both hypomagnesemia and hypophosphatemia are common, as seen in our patient. ECG findings include those consistent with hypokalemia (decreased T-wave amplitudes, U-waves, and widened QRS-complexes), but also sinus tachycardia, 1st degree AV block and arrhythmias, including atrial and ventricular fibrillation [3].

While not completely understood, TPP is thought to be caused by skeletal muscle ion channel dysfunction. Varying degrees of hypokalemia are seen, with the severity often correlating with the degree of muscle weakness [2]. The inwardly rectifying potassium channel Kir2.6, which contributes to the resting potential of mainly skeletal muscle, has been linked to TPP, with up to 33% of patients in certain populations having mutations in its gene, KCNJ18. Notably, the gene has a thyroid response element in its promoter, suggesting that thyrotoxicosis would alter its transcription [5]. Skeletal muscle Na/K ATPase overactivity has also been suggested, leading to an excessive intracellular shift of potassium and inexcitability of muscle fibers. Thyroid hormone increases Na/K ATPase activity both by itself and also indirectly by increasing sensitivity to beta-adrenergic stimulation [1]. It has been hypothesized that patients with TPP have mild ion channel defects which only become significant enough to cause symptoms in the setting of thyrotoxicosis, through the mechanisms described above [6]. In addition, insulin and testosterone are both thought to act synergistically to increase Na/K ATPase activity, with the latter providing a potential explanation for why TPP is seen almost exclusively in males [4]. One study [7] found that patients with TPP had higher levels of testosterone and an increased release of insulin in response to glucose as compared to hyperthyroid patients without TPP, further pointing to the contributory roles of these hormones. Episodes of TPP are sometimes triggered by intense exercise and high-carbohydrate meals, which could be explained by increased levels of beta-adrenergic agonists and insulin, respectively [1, 4]. With this in mind, it is interesting to note that our patient had a physically strenuous job and would regularly experience leg weakness after coming home from work in the weeks leading up to his admission.

Our patient presented with a history of back pain and leg weakness suggestive of nerve root or spinal compression, but no signs of either were seen on a CT of the lumbar spine. While myalgia is not a hallmark of TPP, it does occur and can be misleading. Our patient was noted to be weak in his upper extremities as well. While other causes for acute quadriparesis (e.g., myasthenia gravis or Guillain–Barré syndrome) must be considered, concurrent hypokalemia should alert the clinician to consider TPP and hypokalemic periodic paralysis, a hereditary, more common diagnosis which presents virtually identically. Thyroid function tests should be obtained to distinguish between these two conditions. Muscle weakness in TPP can range from mild to severe and, rarely, life-threatening due to respiratory compromise. Potassium repletion shortens the time to recovery, with muscle strength usually returning to normal once normokalemia is achieved. A maximum of 90 mEq oral potassium is recommended per 24-hour period to prevent rebound hyperkalemia, which is seen in around half of patients. Refractory hypokalemia can be treated with nonselective beta-blockers (e.g., propranolol), which may also be beneficial for potential concurrent symptoms of thyrotoxicosis [3]. Cardiac monitoring is recommended during treatment since fatal arrhythmias (e.g., sinus arrest and ventricular fibrillation) secondary to hypokalemia have been described, although rare. Any cause of thyrotoxicosis, including exogenous hyperthyroidism, can cause TPP. Graves' disease, as seen in our patient, is the most common underlying disorder. Once stabilized, the goal is to investigate the etiology of thyrotoxicosis and provide appropriate treatment since maintaining a euthyroid state will prevent further episodes of TPP [1].

## 4. Conclusion

Thyrotoxic periodic paralysis is a muscular disorder which can complicate thyrotoxicosis of any etiology. Due to its rare nature, a high index of suspicion is required for diagnosis in patients who present with muscle weakness, hypokalemia and symptoms of thyrotoxicosis. Patients need to be closely monitored due to the risks of respiratory failure and cardiac arrhythmias. Acute episodes will resolve with restoration of normokalemia, while treatment of the underlying cause of thyrotoxicosis will prevent recurrence.

## Figures and Tables

**Table 1:** Initial laboratory investigations.

Lab	Value	Reference	Units
Potassium	1.7	3.5–5.0	mmol/L
Magnesium	1.3	1.5–2.2	mEq/L
Phosphorus	1.5	2.6–4.9	mg/dL
TSH	0.010	0.450–5.330	mIU/ml
Free T4	2.98	0.58–1.64	ng/mL
Total T3	399	76–181	ng/dL
TSI	278	<140	% of baseline
Thyroglobulin antibodies	20.0	≤1	IU/mL
Thyroid peroxidase antibodies	180.0	<9	IU/mL

## Data Availability

No data were used to support this study.
